# Association between ancestry and tumor somatic mutations in a large national cohort of women with breast cancer

**DOI:** 10.1038/s41523-025-00815-x

**Published:** 2025-10-29

**Authors:** Kirkpatrick B. Fergus, Justin Newberg, Ray Greenstein, Laura Fejerman, Luis Carvajal-Carmona, Eric Collisson, Niharika Dixit, Garrett Frampton, Franklin W. Huang, Susan L. Neuhausen, Elad Ziv

**Affiliations:** 1 Department of Surgery, University of California, San Francisco, San Francisco, CA USA; 2 Foundation Medicine, Boston, MA USA; 3 Division of Epidemiology, Department of Public Health Sciences, University of California, Davis, CA USA; 4 Department of Biochemistry Molecular Medicine, University of California, Davis, Davis, CA USA; 5 Division of Human Biology, Fred Hutch Cancer Center, Seattle, WA USA; 6 Division of Hematology and Oncology, Department of Medicine, University of California, San Francisco, San Francisco, CA USA; 7 Division of Hematology and Oncology, Zuckerberg San Francisco General Hospital, San Francisco, CA USA; 8 San Francisco Veterans Affairs Medical Center, San Francisco, CA USA; 9 Chan Zuckerberg Biohub, San Francisco, CA USA; 10 Weill Cancer Hub West, San Francisco, CA USA; 11 Institute for Human Genetics, University of California, San Francisco, CA USA; 12 Beckman Research Institute of City of Hope, Duarte, CA USA; 13 Division of General Internal Medicine, Department of Medicine, University of California, San Francisco, San Francisco, CA USA; 14 Helen Diller Family Comprehensive Cancer Center, University of California, San Francisco, San Francisco, CA USA

**Keywords:** Cancer genomics, Breast cancer

## Abstract

Somatic mutations and copy number alterations in breast tumors are important to determine prognosis, predict treatment response, and identify targets for therapy. We utilized somatic sequencing data of breast tumors from Foundation Medicine Inc. to evaluate the association between genetic ancestry and somatic mutations. We used germline variants to infer genetic ancestry with both principal components analysis and ADMIXTURE. Overall, we identified 91 ancestry-specific somatic differences across 58 unique genes, which included potentially targetable genes such as *PIK3CA* found in higher frequency in European ancestry, and *EGFR* found in higher frequency in East Asian ancestry. Pan-cancer analysis of East Asian ancestry and *EGFR* also found higher frequency in prostate, thyroid, and kidney cancers. African ancestry was associated with increased frequency of copy number alterations overall and decreased frequency of multiple genes on the PI3K-AKT pathway. Future research is warranted to elicit the genetic and environmental conditions that underly these findings.

## Introduction

Tumor genomics, including somatic mutations and copy number alterations, is increasingly leveraged in precision medicine for the treatment of cancer^1,2^. A variety of specific mutations help to determine prognosis^3^ and predict treatment response to traditional chemotherapy^4^. Physicians are even parlaying insights from discovered somatic mutations into the design of targeted therapies, such as those for *PIK3CA*, *EGFR*, and *NTRK*^2,5–7^.

Race/ethnicity, as well as genetic ancestry, has been associated with a range of somatic mutations in cancer^8^, and differences in mutation epidemiology in the setting of racial/ethnic clinical trial enrollment disparities are an important health equity consideration in the modern era of precision medicine^9,10^. For example, East Asian ancestry is associated with *EGFR* mutations that drive non-smoking derivative cases in lung cancer^11^, whereas smoking is more commonly associated with *KRAS* mutations^12^. To identify new associations, we and others^13^ recently evaluated tumor mutational profiles in relation to ancestry in The Cancer Genome Atlas (TCGA) and found several copy number alterations and somatic mutations associated with ancestry^14^. Other studies have used small sample sizes of specific ancestry groups to compare to TCGA somatic mutation frequencies^15,16^. However, the modest absolute number of individuals with non-European ancestry in TCGA limited the power of these studies to find associations, particularly with survival or other important clinical factors. Moreover, the ability to detect important clinical differences is limited by the availability of genetic data from diverse populations^17^.

Industry has helped to increase data availability in all populations for breast cancer, the most common malignancy in women and the second leading cause of cancer-related death in the United States^18^. However, the degree to which breast cancer somatic mutations and copy number alterations differ among populations of different ancestries is not well-known. Here, we used a large database of 40,425 breast tumors, inferred their genetic ancestry, and then analyzed associations with short variant mutations and copy number alterations in 287 genes.

## Results

### Genetic ancestry

**Fig. 1:**
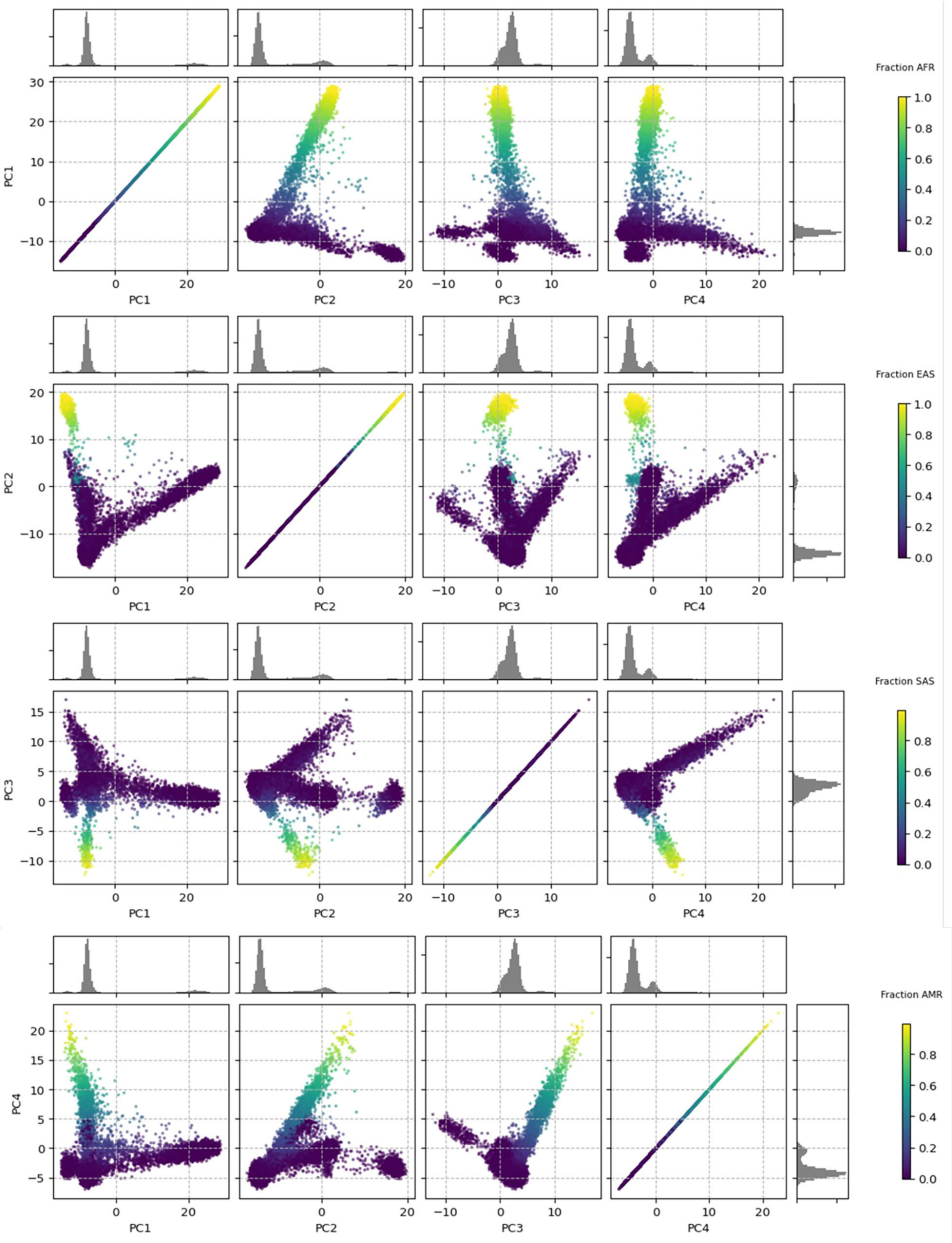
Principal component analysis of Foundation Medicine Inc. cohort. Results of ADMIXTURE for a five-population model projected onto 1000 Genomes data, with ADMIXTURE fraction denoted by color gradient scale. PC principal component, AFR African ancestry, EAS East Asian ancestry, SAS South Asian ancestry, AMR Indigenous American ancestry.

Our sample consisted of 40,425 women with breast cancer. The results of the ADMIXTURE analysis to infer genetic ancestry on a continuous scale using a five-population model are shown in Fig. 1. After categorizing individuals based on their predominant ancestry (>50%), the largest ancestry group was European, representing ~77% of the study sample (Table 1). The second largest group was women with >50% African ancestry, which was ~13.8% of the sample. We also observed 1350 (3.3%), 777 (1.9%), and 434 (1.1%) individuals with >50% East Asian, Indigenous American, and South Asian ancestries, respectively. For 1031 (2.6%) participants, there was no predominant ancestry (>50%).

**Table 1:** Demographic and health characteristics (*n* = 40,425)

	*N*
Demographic characteristics	
Ancestry^a^ *n*(%)	
African	5573 (13.8)
European	31,260 (77.3)
East Asian	1350 (3.3)
South Asian	434 (1.1)
Indigenous American	777 (1.9)
Non-dominant	1031 (2.6)
Age mean (SD)^b^	58.1 (12.8)
Metastatic disease %	
Distant	18,891 (46.7)
Lymph node	4776 (11.8)
Local invasion	13,445 (33.3)
Ambiguous	3239 (8.0)
Unknown	74 (0.2)
TMB^c^ %	4.5 (8.2)
ER positive^d^ %	
None	36,248 (89.7)
Negative	1590 (3.9)
Positive	2587 (6.4)
HER2 positive^e^ %	
Negative	91.5
Positive	8.5

^a^Ancestry defined by ADMIXTURE analysis, with patients categorized by >0.5 proportion of a given ancestry group, otherwise categorized as “nondominant”.

^b^*SD* standard deviation.

^c^*TMB* tumor mutational burden, *TMB* statistic calculated as the number of somatic, coding. mutations (base substitutions and indels) per megabase of genome examined, excluding non-coding regions.

^d^*ER* estrogen receptor.

^e^*HER2* human epidermal growth factor 2.

Principal component analysis (PCA) was performed as a complementary method to inform ancestry. To help inform the interpretation of the PCA results, we first performed PCA using the 1000 Genomes reference samples and projected the PC’s from the 1000 Genomes dataset for each woman in the Foundation Medicine Inc. (FMI) dataset (Supplemental Fig. 1). Overall, the results from the PCA were in high agreement (Fig. 1). The first principal component captured an African vs. non-African ancestry cline, which is consistent with prior studies and likely identifies the out-of-Africa population bottleneck that occurred in human demographic history. The second PC captured women with high East Asian ancestry. The third PC separated women of South Asian ancestry from those of Indigenous American ancestry. The fourth captured increasing Indigenous American ancestry.

### Estrogen receptor hormone status

In the FMI data with available estrogen receptor (ER) status by immunohistochemistry (IHC), 62% of tumors were ER+. Because ER-status is strongly associated with many somatic events, we sought to develop a predictor of ER-status to adjust for in tests of association with ancestry in subsequent analyses. Therefore, we built a multi-gene predictor model of ER-status with a separate, previously published dataset (MSK-IMPACT)^19^. Our final model included 16 genes (Supplemental Fig. 2). The results of applying the model in the validation subset of MSK-IMPACT showed an area under the receiver operator characteristic (AUROC) of 0.83 for ER-probability. The AUROC for applying the same model to the FMI dataset with data on ER-status by IHC was ~0.81.

We performed association analyses between copy number alterations, somatic mutations, and ancestry in the full dataset using ancestry as a continuous variable. A total of 91 associations between tumor somatic mutations and ancestry were identified across 58 unique genes (Supplementary Data 1a–e and 2a–e). The majority of the associations were copy number alterations (*n* = 54), and the remainder were short variant mutations (*n* = 37). Only four genes (*MCL1*, *NTRK1*, *PIK3CA*, *RB1*) had both copy number alterations and short variant mutations. The most common tumor somatic mutations in our total sample included *TP53* (*n* = 20,734) and *PIK3CA* (*n* = 14,066). The most common amplification was at the *MYC* locus (*n* = 7970). In this section, we highlight the most frequent somatic mutations in breast cancer and investigate associations with ancestry groups, as well as associations with predicted ER-status.

### African ancestry

**Fig. 2:**
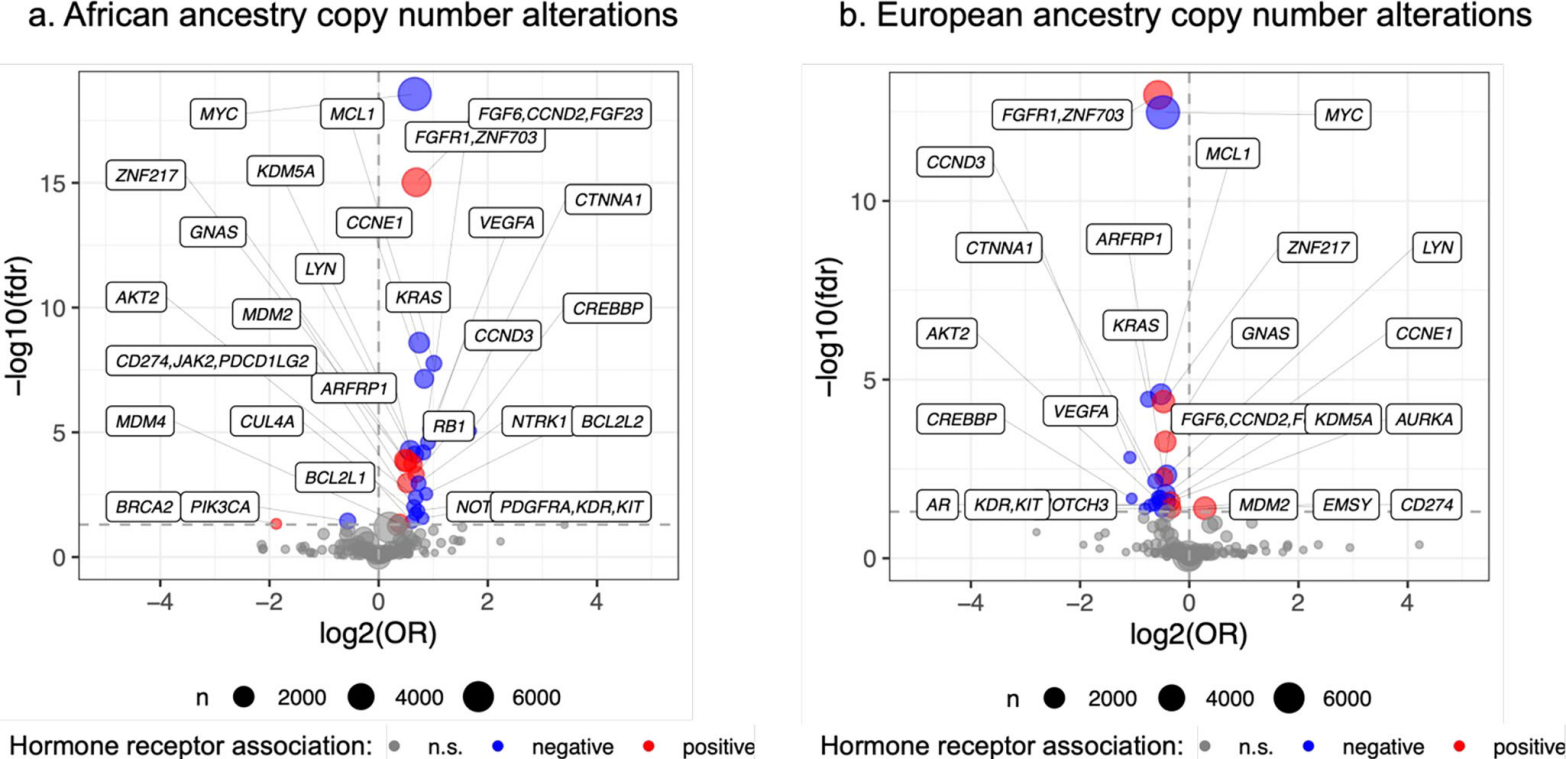
Tumor somatic copy number alteration associations with ancestry. Volcano plots demonstrating results of logistic regression between copy number alterations and African ancestry (**a**) and European ancestry (**b**) as continuous variables. The x-axes represent the odds ratio (OR) and y-axes represent the false discovery rate (FDR). FDR significance is denoted by the horizontal dotted line.

Compared with other ancestry groups overall, we found a relatively higher prevalence of copy number alterations in breast cancer patients of African ancestry (Fig. 2a), most associated with ER-negative breast cancer. In contrast, only two copy-number alterations were less frequent in this population after false discovery rate (FDR) correction (*BRCA2* and *PIK3CA*). The two most common and highly significant copy number alterations seen in this ancestry group were *MYC* (*n* = 1528; odds ratio (OR) = 1.58; FDR = 2.75 × 10^−19^), which was associated with ER-negative breast cancer, and *FGFR1* (*n* = 889; OR = 1.62; FDR = 9.48 × 10^−16^), which was associated with ER-positive breast cancer. We also noted that African ancestry was associated with a higher frequency of copy number alterations in *CD274/JAK2/PDCD1LG2* (*n* = 150; OR = 1.56; FDR = 0.01), which was preferentially found in ER/HER2-negative breast cancer.

**Fig. 3:**
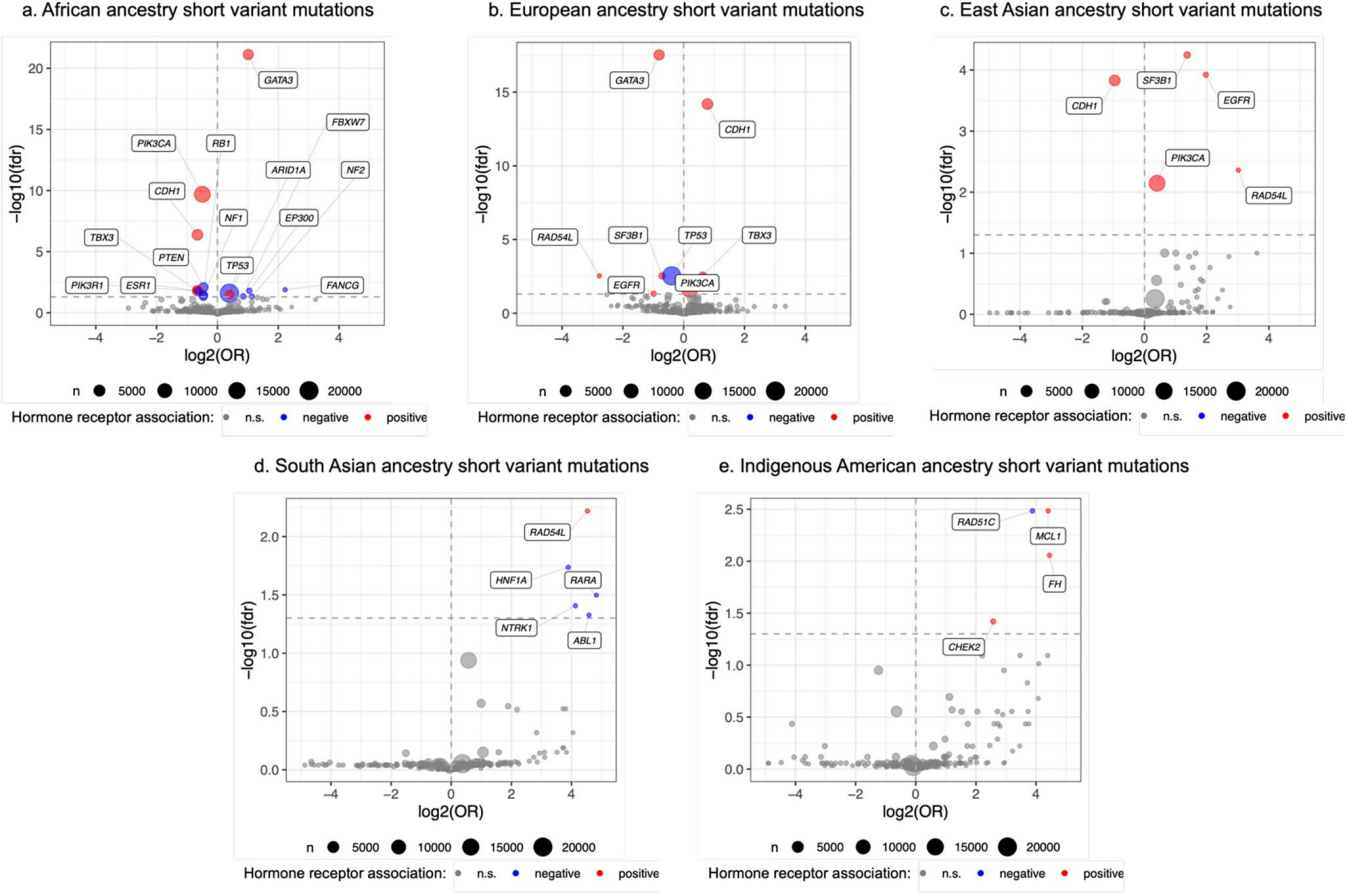
Short variant mutations associated with ancestry. Volcano plots demonstrating results of logistic regression between short variant mutations and African ancestry (**a**), European ancestry (**b**), East Asian ancestry (**c**), South Asian ancestry (**d**) and Indigenous American ancestry (**e**). The x-axes represent the log odds ratios (OR) and y-axes represent the false discovery rate (FDR). FDR significance is denoted by the horizontal dotted line.

The most common somatic short variant mutation observed at a higher frequency among those with African ancestry in our sample was *TP53* (*n* = 3512; OR = 1.31; FDR = 0.025) (Fig. 3a), which was associated with ER-negative breast cancer. For ER-positive breast cancer, we noted a highly statistically significant association with *GATA3* (*n* = 654; OR = 2.02; FDR = 7.52 × 10^−22^).

### European ancestry

Among those of European ancestry, we observed a lower frequency of copy number alterations overall (Fig. 2b). The most significant were a lower frequency of mutations in *MYC* (*n* = 5179; OR = 0.72; FDR = 3.32 × 10^−13^), associated with ER-negative breast cancer, and *FGFR1* (*n* = 3716; OR = 0.67; FDR = 1.08 × 10^−13^), associated with ER-positive breast cancer.

Having a majority fraction of European ancestry was associated with lower frequencies of short variant mutations, most commonly in *TP53* (*n* = 13,921; OR = 0.76; FDR = 0.003), associated with ER-negative breast cancer (Fig. 3b). The most common mutation observed at higher frequencies was *PIK3CA* (*n* = 10,659; OR = 1.14; FDR = 0.024), followed by *CDH1* (*n* = 3767; OR = 1.72; FDR = 6.49 × 10^−15^), both associated with ER-positive breast cancer.

### South and East Asian ancestry

No significant associations were observed between either South Asian or East Asian ancestry and copy number alterations in this breast cancer cohort. Among tumors from women of East Asian ancestry, short variant associations in several genes were found at higher frequencies, most notably in *EGFR* (*n* = 22; OR = 3.92; FDR = 0.0001). This and other associations with East and South Asian ancestry are shown in Fig. 3.

### Indigenous American ancestry

Indigenous American ancestry was associated with a higher frequency of *BRCA1* (*n* = 20; OR = 6.9; FDR = 0.02). We also found a higher frequency of four short variant mutational signatures (Fig. 3e), most notably *RAD51C* (*n* = 11; OR = 14.73; FDR = 0.003) associated with ER-negative breast cancer and *CHEK2* (*n* = 184; OR = 5.96; FDR = 0.04) associated with ER-positive breast cancer.

### PI3K-AKT pathway mutations

Our results identified numerous genes along the PI3K-AKT pathway with mutations found in lower frequency among those of African ancestry. Most notably, potentially targetable short variant mutations in *PIK3CA* (*n* = 1397; OR = 0.71; FDR = 2.04 × 10^−10^) were found less frequently among those with African ancestry. We also observed fewer copy number alterations in *PIK3CA* (*n* = 114; OR = 0.67; FDR = 0.036) in this group. Thus, we examined other genes along the pathway, including *PTEN* (*n* = 350; OR = 0.73; FDR = 0.008) and *PIK3R1* (*n* = 163; OR = 0.64; FDR = 0.016), both of which were less frequent with similar odds ratios. AKT mutations had mixed results, with non-significant associations for *AKT1* short variant mutations (*n* = 182; OR = 0.82; FDR = 0.38) and copy number alterations (*n* = 43; OR = 0.68; FDR = 0.32). In contrast, a higher frequency of *AKT2* copy number alterations was observed in our sample population (*n* = 148; OR = 1.6; FDR = 0.004). Notably, while most of these mutations were associated with ER-positive disease, *PTEN* was associated with ER-negative disease, suggesting that the association was not simply due to a lower frequency of ER-positive disease in African Americans.

### CREBBP deletion and EP300 mutation

Another pathway we investigated that was associated with African ancestry was *CREBBP/EP300*. Specifically, copy number deletions in *CREBBP* (*n* = 56; OR = 3.23; FDR = 8.73 × 10^−6^) were seen at higher frequencies in this group. In addition, short variant mutations in the gene *EP300*, which encodes for a histone acetyltransferase homologue of *CREBBP*, were also associated with African ancestry (*n* = 59; OR = 2.07; FDR = 0.016).

### *EGFR* mutations across cancer types

**Fig. 4:**
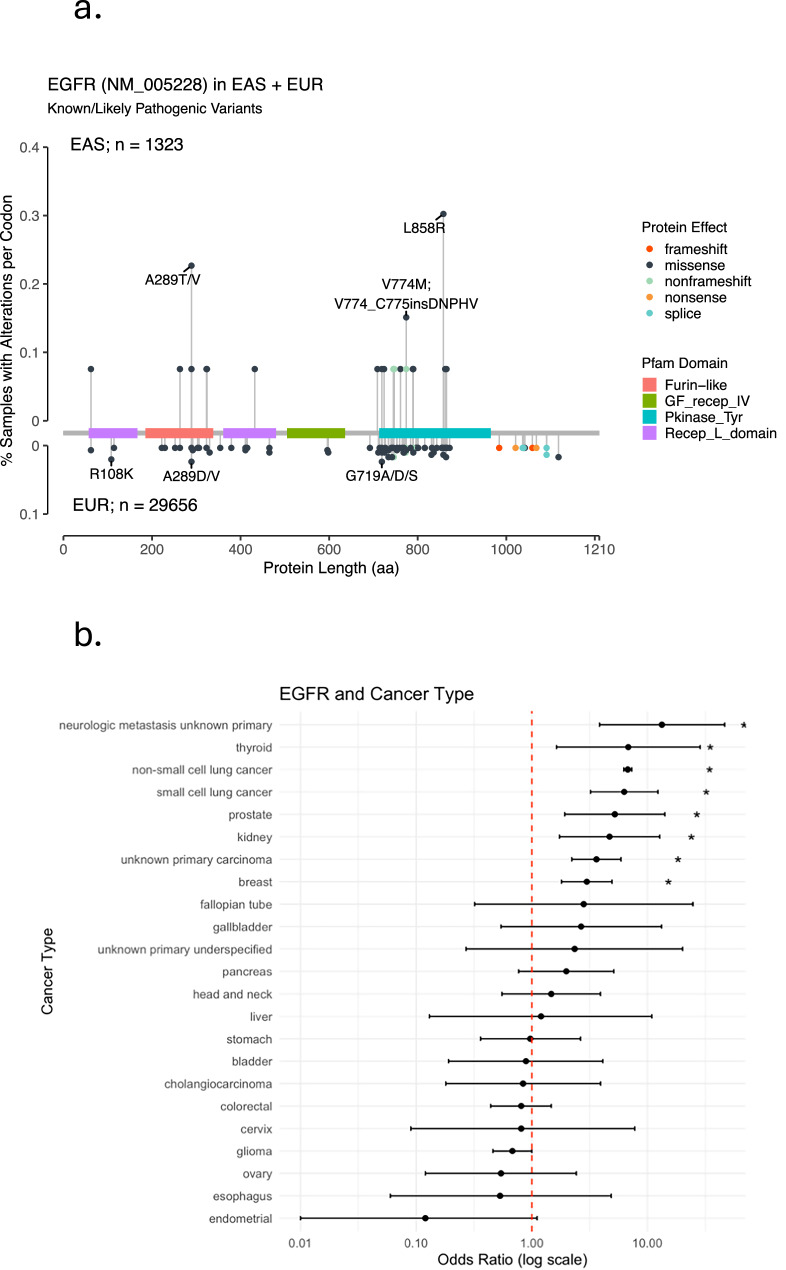
Associations between EGFR mutations and East Asian ancestry. **a** Lollipop diagram of *EGFR* mutations among those with East Asian and European ancestry demonstrating pathogenic and likely pathogenic variants. Figure includes women and men with breast cancer in the Foundation Medicine database. *EGFR* Epidermal growth factor receptor, EAS East Asian ancestry, EUR European ancestry. **b** Forrest plot of the association between East Asian ancestry and EGFR among other cancer types available in the FMI databank. Non-breast cancer samples from FMI are processed in the same manner as the breast cancer samples described in the methods. Odds ratios and 95% confidence intervals from logistic regression controlling for testing platform and tumor mutational burden. Data shown on logarithmic scale. * = false discovery rate < 0.05. Not shown are cancer types with standard error > 5. *EGFR* Epidermal growth factor receptor.

To further investigate the novel association between East Asian Ancestry and *EGFR* somatic mutations, we first examined the frequency of these rare mutations (Fig. 4a). We noted that a subset of these were activating mutations such as L858R. We subsequently sought to analyze other tumor types in the pan-cancer FMI databank (Fig. 4b), due to the already known association between *EGFR* mutations and lung cancer in this population. While *EGFR* mutations in non-small cell lung cancer were by far the most common and statistically significant in this population (*n* = 1630), we also found significant associations with small cell lung cancer, prostate cancer, kidney cancer, thyroid cancer, and the heterogeneous group of unknown primary cancers. Notable null findings, though not an exhaustive list, were observed for glioma, pancreatic cancer, head and neck cancer (excluding thyroid), colorectal cancer, and stomach cancer. As a sensitivity analysis, we examined the substrata of breast and prostate cancer samples collected directly from the primary site only, and the association remained statistically significant (FDR < 0.01) with increased effect size for both the breast (OR = 3.71; 95% confidence interval (CI) 1.66–8.33) and prostate (OR = 9.21; 95% CI 3.28–25.89).

## Discussion

We evaluated the largest database of sequenced breast cancer tissue samples, including >40,000 women, for associations between somatic mutations and ancestry. Our study offers important insights into breast cancer somatic mutations for non-European ancestry groups^8^. We found 91 associations between genetic ancestry and somatic mutations that were significantly different based on either short variant mutation frequency or copy number alteration, pointing to some established as well as some novel potential biological mechanisms that differ according to genetic ancestry. Importantly, we observed a few examples of multiple genes in the same pathway associated with ancestry, as well as a case of *EGFR* mutations seen across multiple cancer types in the same ancestry group.

One of the key findings of our large cohort analysis was that breast cancer *EGFR* mutations were more common among women of East Asian ancestry, although these mutations were rare in all populations. Prior studies have shown associations between *EGFR* mutations and poor breast cancer prognosis^20,21^. The association between East Asian ancestry and *EGFR* mutation status is consistent with the well-known observation of comparatively high *EGFR* mutations in lung cancer in this population^11^. While prior breast cancer studies have noted a high rate of *EGFR* mutations in study samples with predominantly Asian participants^22^ compared to predominantly European participants^23^, there have been no studies that systematically compared frequencies in the same dataset that have been large enough to demonstrate this difference with a high degree of significance. The *EGFR* mutations seen in our cohort included mutations that are considered activating mutations, suggesting that the gene may act as a driver in these tumors. Thus, although rare, these mutations may offer a target for therapy in women with TNBC, particularly among women of East Asian ancestry.

This excess in *EGFR* mutations was consistent in multiple other cancer types (though not all types), including lung, breast, prostate, kidney, and unknown primary carcinomas. The point estimate for the odds ratio with East Asian ancestry was consistent for most of these cancers. Prior studies have shown worse prognosis among those with *EGFR* overexpression in prostate cancer^24^, including associations with hormone-refractory prostate cancer^25^, and increased recurrence/relapse after prostatectomy^26^. While some studies investigating the association between *EGFR* mutations and self-reported race were negative^27^, these were likely underpowered to identify differences in genes with rare mutation frequencies such as *EGFR*. Interestingly, one case study revealed a rare germline *EGFR* variant in a Chinese family with two prostate cancer cases associated with biallelic *CDK12* inactivation^28^.

In summary, our study confirms a previously known association between *EGFR* somatic mutations and East Asian ancestry, and is the first to systematically demonstrate a similar association in breast, prostate, and kidney cancers. Our findings suggest that while tissue/cell type is the major determinant of somatic mutations, genetic and/or environmental factors associated with genetic ancestry may modify the frequency of somatic mutations across cancer types. Prior studies have shown that HER2 amplification in breast cancer is more common among Asian women and among Latin American women with higher Indigenous American ancestry^29,30^. Since *ERBB2* is a member of the *EGFR* family, it is possible that the same risk factors underlying this higher prevalence affects multiple members of the same pathway.

Our results indicate that the majority of African-ancestry differences were seen in copy number alterations, which have previously been reported in the FMI data for breast cancer^31^. African American women are at increased risk of triple negative breast cancer, which has a higher prevalence of somatic signatures associated with homologous recombination repair deficiency^16,32^. Since these tumors tend to have higher genomic instability overall, it is possible that this may explain the higher prevalence of copy number alterations. A higher prevalence of copy number alterations has also been observed in prostate tumors from men of higher African ancestry in the FMI dataset^33^.

Mutations in *PIK3CA* were significantly less common among women with higher African ancestry, as has been reported before^34,35^. We also found that none of the *PIK3CA* hot spots were consistently associated with ancestry. *PTEN* and *PIK3R1* were also significantly lower among women with higher African ancestry. These associations among women of African ancestry suggest that the underlying factor that decreases *PIK3CA* mutation frequency could affect the entire PI3K-AKT pathway. As *PTEN* mutations are preferentially associated with ER-negative breast cancer, it is unlikely that this association is due to the lower prevalence of ER-positive breast tumors in women of African ancestry. Since there are multiple drug targets in this pathway, including *PIK3CA* inhibitors, which are approved for breast cancer, and *AKT1* inhibitors, the lower prevalence of mutations in this pathway may lead to worsening disparities in outcomes, as these treatments would not be efficacious in this group.

We also found more frequent *EP300/CREBBP* mutations among individuals of African ancestry. These genes encode for two related histone acetyltransferases with similar functions that act as co-activators in transcriptional regulation, predominantly through the acetylation of proteins^36^. Short variant mutations in *EP300* and *CREBBP* copy number deletions are more likely to be found in ER-negative breast cancer. Prior functional genomics studies have shown that *CREBBP* is a candidate driver of aggressive triple negative breast cancer, with *CREBBP* proteins absent in 8% of tissue samples^37^. Given the poor prognosis of triple negative breast cancer as well its association with those of African descent^34^, our findings highlight a potentially important driver of this breast cancer subtype for those with African ancestry. It is notable that the *EP300*/*CREBBP* pathway mutational signatures uncovered in this study are also seen in diffuse large B-cell lymphoma, where they are associated with *NOTCH* activation via negative regulation of *FBXW7*^38^. Future research is required to investigate whether this phenomenon is applicable in breast cancer.

Our study also found higher frequencies among individuals of African ancestry of copy number alterations in *CD274/JAK2/PDCD1LG2*. This copy number alteration is generally considered rare^39^. However, a prior small sample study of metastatic TNBC patients with *CD274* copy number amplification found that this somatic mutation was associated with better overall survival during immune checkpoint inhibitor therapy^40^. Our finding that African ancestry is associated with higher frequencies of these rare mutations necessitates further research on this biological mechanism and how this patient population that carries a disproportionate burden of TNBC could stand to benefit from novel immune checkpoint inhibitor treatments.

Overall, our findings indicate that genetic ancestry is correlated with somatic mutations and copy number alterations in breast cancer. Our study has many strengths, including leveraging one of the largest cohorts of sequenced breast cancer tumors to date. Importantly, while individuals of non-European ancestry comprise a minority of the sample, the sample size remains large and powered to detect differences in mutation frequency within minority groups, which to date remains under-investigated. In particular, our study included over 5000 women with >50% African ancestry, which should substantially increase the knowledge about somatic events in breast cancer in women of this ancestry. Our study has more limited sample size to infer events in women of East Asian ancestry. For both South Asian and Indigenous American ancestries, our study included <1000 women, and thus our conclusions are most limited in those ancestries. Larger studies in these populations will be required to identify any differences in somatic mutations.

Our estimation of ancestry used >50,000 SNPs, and we found consistency between PCA and ADMIXTURE. Other studies using similar numbers of SNPs also developed robust predictors of continental ancestry^41^, and prior studies of FMI data have also identified associations with African ancestry in other tumor types^31,33^. However, subcontinental ancestry (e.g., different regions of East Asia) was not ascertained, and our ability to infer ancestries that are more closely related may be limited. An important limitation to our study is that genetic ancestry captures information about inherited genetic variants, but cannot assess the impact that race, a social construct, may place in upstream environmental and social exposures. Somatic mutations may be associated with environmental exposures, such as KRAS in lung cancer with smoking^42^. Furthermore, there may be biases by insurance coverage and provider preference that affect which patients have tumors that are sent for genomic analysis. Our study cannot easily differentiate between these determinants, and future studies with information about both germline genetic information and social and environmental exposures are needed to assess the etiology of these associations.

In addition, use of targeted sequencing will not capture genes unknown to be drivers of cancer. Thus, more whole-exome and whole-genome discovery studies may be needed to identify genes recurrently mutated in cancer in different ancestry groups. We also had limited IHC testing or annotation availability, which impacts our ability to identify ER-positive and negative disease. If we inadequately adjusted for the association between ER-negative breast cancer and African ancestry, we would have over-detected associations with genes recurrently mutated in ER-negative BC but under-detected associations between African ancestry and genes recurrently mutated in ER-positive breast cancer. Therefore, the associations we report with ER-negative breast cancer genes and African ancestry should be taken with caution, but the associations we reported with ER-positive related breast cancer genes (e.g., GATA3, FGFR1) are likely correct. Nevertheless, our study provides several avenues worthy of further investigation, including the role of *EGFR* in East Asian populations with cancer and the association between ancestry and somatic mutations across multiple genes in the same pathway.

## Methods

### Study sample

This study included data from patients with local or metastatic breast cancer with tissue samples submitted to FMI for Comprehensive Genomic Profiling (CGP) by the FoundationOne® (F1) and FoundationOne®CDx (F1CDx) platforms. The patient samples were de-identified, and consent was obtained for research. Clinical samples were collected between 2013 and 2022 throughout the United States. Formalin-fixed paraffin-embedded (FFPE) tissue specimens were sent to FMI. CGP was used to sequence samples and assess genetic variance as discussed below. Pan-cancer FMI tissue samples of primary origin outside the breast used in our sub-analysis were collected and processed by FMI in the same time period, with a similar nationwide catchment area.

### Experimental model and study participant details

Approval for this study, including a waiver of informed consent and Health Insurance Portability and Accountability Act waiver of authorization, was obtained from the Western Institutional Review Board (Protocol #20152817) and guided by principles outlined in the Declaration of Helsinki. The Institutional Review Board granted a waiver of informed consent under 45 CFR § 46.116 based on review and determination that this research meets the following requirements: (i) the research involves no more than minimal risk to the subjects; (ii) the research could not practicably be carried out without the requested waiver; and (iii) the waiver will not adversely affect the rights and welfare of the subjects. We also used the Memorial Sloan Kettering Cancer Center cohort data (MSK IMPACT)^19^ which was publicly available and approved by their Institutional Review Board, and all patients provided written informed consent for tumor sequencing and review of patient medical records for detailed demographic, pathologic, and treatment information (NCT01775072). All samples were obtained during routine clinical care.

### Genomic sequencing

Genomic sequencing methods have been described previously^31,43,44^. In brief, FMI’s in-house laboratory has been certified by Clinical Laboratory Improvement Amendments (CLIA) and accredited by the College of American Pathologists. F1CDx^43^ adapted methods and technology from its predecessor, F1, which targeted 287 cancer-related genes^44^. F1CDx used ~50–1000 ng of DNA that was extracted from FFPE patient samples for whole-genome shotgun library construction. Next-generation sequencing (NGS) was performed using an Illumina® platform with hybrid capture targeting 324 cancer-related genes, aiming for a median coverage of >500× and >99% of exons covered at >100× depth. Only variants in the cancer-related genes of interest underwent variant calling for both F1 and F1CDx. FMI’s proprietary software then analyzed sequence data, and quality control consisted of duplicate read removal, sequencing metrics checks with Picard and SAMtools^45^, and use of proprietary FMI variant call quality filters that are tuned for expected intrinsic sample noise^43^. Variant calls passing quality control metrics were then classified as short variants (which were mostly point mutations classified according to COSMIC designations but also included indels and truncating variants in tumor suppressor genes that span a single baiting interval and thus are more generally small mutations) or copy number alterations (amplifications and homozygous gene deletions) identified with comparative genomic hybridization methods^46^. As has been described previously^47^, likely germline variants were filtered out prior to final somatic classification. For the germline ancestry analyses, we selected variants without regard to likely function.

### Ancestry determination

Patient ancestry was calculated using ADMIXTURE. The ancestry groups were defined by using the 1000 Genomes Project (IGSR), version 3^48^. We used all of the samples from IGSR for ADMIXTURE and PCA. These included 836 individuals of African (AFR) ancestry, 601 individuals of East Asian, 658 individuals of European (EUR) ancestry, 497 individuals of Mixed American (AMR), and 522 individuals of South Asian (SAS).

Over 50,000 single-nucleotide polymorphisms (SNPs) present in both IGSR and CGP tests were used in this analysis. To infer ancestry using a model, we entered data from Phase 3 of IGSR into ADMIXTURE (version 1.3)^49^, using the unsupervised setting. We used K = 5 signatures to produce a P-file that captured population allele frequencies. Although previous studies have tested ADMIXTURE with larger Ks (for example, *K* = 8 was used in Fig. 2 of the seminal IGSR Nature paper^48^), we found that with our SNP sets, *K* = 5 produced interpretable and consistent signatures. To interpret the ancestries, we labeled the ancestry that was dominant in each of the super-populations from IGSR as follows: the AFR super-population as African, in EAS super-population as East Asian, the EUR super-population as European, and the SAS super-population as South Asian. To label Indigenous American ancestry, we used the component of ancestry that was present only in the AMR population, but this component was not dominant in this population, as individuals in this population had a broad range of admixture, including European and African ancestry, as has been previously reported^48^. CGP samples were prepared for ADMIXTURE by imputing missing SNPs with Beagle (version 5.0)^50,51^, and then run through ADMIXTURE in projection mode, utilizing the P-file generated from the IGSR reference dataset. This produced a Q-file containing the ancestry fractions of the *K* signatures. For patients with max(fraction) > 0.5, they were assigned an ancestry corresponding to the max(fraction); otherwise, they were classified as not belonging to a group. All individuals were included in all analyses; however, for display of mutational frequencies by ancestry group (“lollipop plots”), we used the groups based on max(fraction)>0.5.

We also ran principal components analysis (PCA) to infer ancestry. The IGSR SNP data were converted to alternate allele counts, and these were run through PCA (Python 3.9.5 and scikit-learn version 0.19.1) to learn a set of eigenvectors. We performed LD pruning with PLINK. The CGP data were also converted to alternate allele counts (counts of 0 were used for missing SNP data), and these were transformed using the learned eigenvectors, which resulted in principal component features, the top four of which were used as covariates in regression analysis. Of note, both the ADMIXTURE analysis and the PCA were run separately on two different CGP datasets (F1; F1CDx), which had differences in the SNP sets, and we used the overlapping SNPs to create a common gene panel.

### Development of a predictor of estrogen receptor status

ER status is a major determinant of tumor biology and somatic mutational profiles and is also associated with ancestry. Since ER status is associated with ancestry—African ancestry women have higher risk of ER-negative breast cancer^52^—and since we did not have ER status on most of the women, we sought to infer ER status using a model based on the somatic mutations and copy number alterations so that we can adjust for it in our analyses. Therefore, we built a multi-gene predictor of ER-status using a previously published dataset^19^. This dataset included *N* = 1857 women, including 1530 women with ER-positive and 327 women with ER-negative breast cancer status based on immunohistochemistry. Briefly, we divided the dataset into a training (2/3) and validation (1/3) set. We performed univariate association testing between ER-status and each gene copy number or somatic mutation in the training set using logistic regression models. We built a multi-gene predictor by forward step-wise addition of the genes that were significant in single-gene association testing in the training set. We then tested prediction in the 1/3 validation data using the predicted ER-status score from the logistic regression model as a continuous variable. A subset of the FMI data (*n* = 4177) had clinically annotated ER status by IHC and was used to perform external validation of that model.

### Statistical analysis

Association testing for each somatic event was performed using logistic regression models and ancestry as a predictor, adjusting for age, sex, predicted ER status, HER2 status as ascertained by copy number amplification using the FMI dataset, metastasis, tumor mutational burden (TMB), and CGP platform type. Tumor mutational burden was calculated as the number of somatic, coding mutations (base substitutions and indels) per megabase of genome examined, excluding non-coding regions and filtering out likely germline variants and possible driver mutations. We used generalized linear modeling with a binomial family in Python with statsmodels (version 0.14.1). For testing associations between somatic mutations and ancestry in various other cancer types, we used short variant mutations in the models and used logistic regression models as above for breast cancers.

## Supplementary information

Supplementary Information

Supplementary Data 1a

Supplementary Data 1c

Supplementary Data 1d

Supplementary Data 1e

Supplementary Data 2a

Supplementary Data 2b

Supplementary Data 2c

Supplementary Data 2d

Supplementary Data 2e

Supplementary Data 3

## Data Availability

Data were generated by the authors but are not publicly available for declared legitimate reasons. The sequencing data generated in this study are derived from clinical samples. All consented data supporting the findings of this study that can be released are provided within the article and supplementary files. Due to HIPAA requirements, we are not authorized to share underlying sequence data or individualized patient genomic data, which contain potentially identifying or sensitive patient information. Foundation Medicine, Inc. is committed to collaborative data analysis, and it has well-established and widely utilized mechanisms by which investigators can query its core genomic database of >600,000 deidentified sequenced cancers to obtain aggregated datasets. For more information and mechanisms of access to the Foundation Medicine, Inc. data in this study, please contact the corresponding authors or the Foundation Medicine, Inc. Data Governance Council at data.governance.council@foundationmedicine.com. You and your institution will be required to sign a data transfer agreement.

## Code availability

The underlying code for this is not publicly available for proprietary reasons.

## References

[1] Chapman, P. B. et al. Vemurafenib in patients with BRAFV600 mutation-positive metastatic melanoma: final overall survival results of the randomized BRIM-3 study. *Ann. Oncol.* **28**, 2581–2587 (2017). 28961848 10.1093/annonc/mdx339 PMC5834156

[2] Pirker, R. et al. Cetuximab plus chemotherapy in patients with advanced non-small-cell lung cancer (FLEX): an open-label randomised phase III trial. *Lancet* **373**, 1525–1531 (2009). 19410716 10.1016/S0140-6736(09)60569-9

[3] Griffith, O. L. et al. The prognostic effects of somatic mutations in ER-positive breast cancer. *Nat. Commun.* **9**, 3476 (2018). 30181556 10.1038/s41467-018-05914-x PMC6123466

[4] Norquist, B. et al. Secondary somatic mutations restoring BRCA1/2 predict chemotherapy resistance in hereditary ovarian carcinomas. *J. Clin. Oncol.* **29**, 3008–3015 (2011). 21709188 10.1200/JCO.2010.34.2980 PMC3157963

[5] Willis, O. et al. PIK3CA gene aberrancy and role in targeted therapy of solid malignancies. *Cancer Gene Ther.* **27**, 634–644 (2020). 31988478 10.1038/s41417-020-0164-0

[6] Kheder, E. S. & Hong, D. S. Emerging targeted therapy for tumors with NTRK fusion proteins. *Clin. Cancer Res.* **24**, 5807–5814 (2018). 29986850 10.1158/1078-0432.CCR-18-1156

[7] Ibrahim, E. M., Abouelkhair, K. M., Al-Masri, O. A., Chaudry, N. C. & Kazkaz, G. A. Cetuximab-based therapy is effective in chemotherapy-naive patients with advanced and metastatic non-small-cell lung cancer: a meta-analysis of randomized controlled trials. *Lung* **189**, 193–198 (2011). 21424607 10.1007/s00408-011-9286-3

[8] Carrot-Zhang, J. et al. Comprehensive analysis of genetic ancestry and its molecular correlates in cancer. *Cancer Cell* **37**, 639–654. e636 (2020). 32396860 10.1016/j.ccell.2020.04.012 PMC7328015

[9] Guerrero, S. et al. Analysis of racial/ethnic representation in select basic and applied cancer research studies. *Sci. Rep.* **8**, 13978 (2018). 30228363 10.1038/s41598-018-32264-x PMC6143551

[10] Aldrighetti, C. M., Niemierko, A., Van Allen, E., Willers, H. & Kamran, S. C. Racial and ethnic disparities among participants in precision oncology clinical studies. *JAMA Netw. Open* **4**, e2133205–e2133205 (2021). 34748007 10.1001/jamanetworkopen.2021.33205 PMC8576580

[11] Mitsudomi, T. Molecular epidemiology of lung cancer and geographic variations with special reference to EGFR mutations. *Transl. Lung Cancer Res.* **3**, 205 (2014). 25806302 10.3978/j.issn.2218-6751.2014.08.04 PMC4367697

[12] Dogan, S. et al. Molecular epidemiology of EGFR and KRAS mutations in 3,026 lung adenocarcinomas: higher susceptibility of women to smoking-related KRAS-mutant cancers. *Clin. Cancer Res.* **18**, 6169–6177 (2012). 23014527 10.1158/1078-0432.CCR-11-3265 PMC3500422

[13] Yuan, J. et al. Integrated analysis of genetic ancestry and genomic alterations across cancers. *Cancer Cell* **34**, 549–560.e549 (2018). 30300578 10.1016/j.ccell.2018.08.019 PMC6348897

[14] Roelands, J. et al. Ancestry-associated transcriptomic profiles of breast cancer in patients of African, Arab, and European ancestry. *NPJ Breast Cancer* **7**, 10 (2021). 33558495 10.1038/s41523-021-00215-x PMC7870839

[15] Ding, Y. C. et al. Profiling the somatic mutational landscape of breast tumors from Hispanic/Latina women reveals conserved and unique characteristics. *Cancer Res.* **83**, 2600–2613 (2023). 37145128 10.1158/0008-5472.CAN-22-2510 PMC10390863

[16] Ansari-Pour, N. et al. Whole-genome analysis of Nigerian patients with breast cancer reveals ethnic-driven somatic evolution and distinct genomic subtypes. *Nat. Commun.* **12**, 6946 (2021). 34836952 10.1038/s41467-021-27079-w PMC8626467

[17] Spratt, D. E. et al. Racial/ethnic disparities in genomic sequencing. *JAMA Oncol.* **2**, 1070–1074 (2016). 27366979 10.1001/jamaoncol.2016.1854 PMC5123755

[18] Siegel, R. L., Miller, K. D., Wagle, N. S. & Jemal, A. Cancer statistics, 2023. *CA Cancer J. Clin.* **73**, 17–48 (2023). 36633525 10.3322/caac.21763

[19] Razavi, P. et al. The genomic landscape of endocrine-resistant advanced breast cancers. *Cancer Cell* **34**, 427–438.e426 (2018). 30205045 10.1016/j.ccell.2018.08.008 PMC6327853

[20] Viale, G. et al. Invasive ductal carcinoma of the breast with the “triple-negative” phenotype: prognostic implications of EGFR immunoreactivity. *Breast Cancer Res. Treat.* **116**, 317–328 (2009). 18839307 10.1007/s10549-008-0206-z

[21] Tsutsui, S., Ohno, S., Murakami, S., Hachitanda, Y. & Oda, S. Prognostic value of epidermal growth factor receptor (EGFR) and its relationship to the estrogen receptor status in 1029 patients with breast cancer. *Breast Cancer Res. Treat.* **71**, 67–75 (2002). 11859875 10.1023/a:1013397232011

[22] Teng, Y. H.-F. et al. Mutations in the epidermal growth factor receptor (EGFR) gene in triple negative breast cancer: possible implications for targeted therapy. *Breast Cancer Res.* **13**, 1–9 (2011). 10.1186/bcr2857 PMC3219198 21457545

[23] Jacot, W. et al. Lack of EGFR-activating mutations in European patients with triple-negative breast cancer could emphasise geographic and ethnic variations in breast cancer mutation profiles. *Breast Cancer Res.* **13**, 1–9 (2011). 10.1186/bcr3079 PMC3326575 22192147

[24] De Muga, S. et al. Molecular alterations of EGFR and PTEN in prostate cancer: association with high-grade and advanced-stage carcinomas. *Mod. Pathol.* **23**, 703–712 (2010). 20208477 10.1038/modpathol.2010.45

[25] Guérin, O., Fischel, J. L., Ferrero, J.-M., Bozec, A. & Milano, G. EGFR targeting in hormone-refractory prostate cancer: current appraisal and prospects for treatment. *Pharmaceuticals* **3**, 2238–2247 (2010). 27713352 10.3390/ph3072238 PMC4036653

[26] Di Lorenzo, G. et al. Expression of epidermal growth factor receptor correlates with disease relapse and progression to androgen-independence in human prostate cancer. *Clin. Cancer Res.* **8**, 3438–3444 (2002). 12429632

[27] Arenas-Gallo, C. et al. Race and prostate cancer: genomic landscape. *Nat. Rev. Urol.* **19**, 547–561 (2022). 35945369 10.1038/s41585-022-00622-0

[28] Qian, K. et al. A novel germline EGFR variant p.R831H causes predisposition to familial CDK12-mutant prostate cancer with tandem duplicator phenotype. *Oncogene* **39**, 6871–6878 (2020). 32978518 10.1038/s41388-020-01476-9

[29] Telli, M. L. et al. Asian ethnicity and breast cancer subtypes: a study from the California Cancer Registry. *Breast Cancer Res. Treat.* **127**, 471–478 (2011). 20957431 10.1007/s10549-010-1173-8 PMC4349378

[30] Marker, K. M. et al. Human epidermal growth factor receptor 2–positive breast cancer is associated with indigenous american ancestry in Latin American women. *Cancer Res.* **80**, 1893–1901 (2020). 32245796 10.1158/0008-5472.CAN-19-3659 PMC7202960

[31] Jiagge, E. et al. Tumor sequencing of African ancestry reveals differences in clinically relevant alterations across common cancers. *Cancer Cell* **43**, 1963–1971.e3 (2023). 10.1016/j.ccell.2023.10.003 PMC11097212 37890492

[32] Polak, P. et al. A mutational signature reveals alterations underlying deficient homologous recombination repair in breast cancer. *Nat. Genet.* **49**, 1476–1486 (2017). 28825726 10.1038/ng.3934 PMC7376751

[33] Koga, Y. et al. Genomic profiling of prostate cancers from men with African and European ancestry. *Clin. Cancer Res.* **26**, 4651–4660 (2020). 32651179 10.1158/1078-0432.CCR-19-4112 PMC7597977

[34] Ademuyiwa, F. O., Tao, Y., Luo, J., Weilbaecher, K. & Ma, C. X. Differences in the mutational landscape of triple-negative breast cancer in African Americans and Caucasians. *Breast Cancer Res. Treat.* **161**, 491–499 (2017). 27915434 10.1007/s10549-016-4062-y PMC5243212

[35] Chen, J. W. et al. Comparison of PIK3CA mutation prevalence in breast cancer across predicted ancestry populations. *JCO Precis. Oncol.* **6**, e2200341 (2022). 36446041 10.1200/PO.22.00341 PMC9812634

[36] Zhu, Y. et al. The role of CREBBP/EP300 and its therapeutic implications in hematological malignancies. *Cancers* **15**10.3390/cancers15041219 (2023). 10.3390/cancers15041219 PMC9953837 36831561

[37] Peck, B. et al. 3D functional genomics screens identify CREBBP as a targetable driver in aggressive triple-negative breast cancer. *Cancer Res.* **81**, 847–859 (2021). 33509944 10.1158/0008-5472.CAN-20-1822 PMC7611219

[38] Huang, Y.-H. et al. CREBBP/EP300 mutations promoted tumor progression in diffuse large B-cell lymphoma through altering tumor-associated macrophage polarization via FBXW7-NOTCH-CCL2/CSF1 axis. *Signal Transduct. Target. Ther.* **6**, 10 (2021). 33431788 10.1038/s41392-020-00437-8 PMC7801454

[39] Guo, L. et al. PD-L1 expression and CD274 gene alteration in triple-negative breast cancer: implications for prognostic biomarker. *Springerplus* **5**, 805 (2016). 27390646 10.1186/s40064-016-2513-x PMC4916110

[40] Bachelot, T. et al. 128O PDL1/CD274 gain/amplification as a predictive marker of checkpoint blockade inhibitor efficacy in metastatic breast cancer: Exploratory analysis of the SAFIR02-IMMUNO randomized phase II trial. *Ann. Oncol.* **31**, S58–S59 (2020).

[41] Nassar, A. H. et al. Ancestry-driven recalibration of tumor mutational burden and disparate clinical outcomes in response to immune checkpoint inhibitors. *Cancer Cell* **40**, 1161–1172.e1165 (2022). 36179682 10.1016/j.ccell.2022.08.022 PMC9559771

[42] Chapman, A. M., Sun, K. Y., Ruestow, P., Cowan, D. M. & Madl, A. K. Lung cancer mutation profile of EGFR, ALK, and KRAS: meta-analysis and comparison of never and ever smokers. *Lung cancer* **102**, 122–134 (2016). 27987580 10.1016/j.lungcan.2016.10.010

[43] Milbury, C. A. et al. Clinical and analytical validation of FoundationOne®CDx, a comprehensive genomic profiling assay for solid tumors. *PLoS ONE* **17**, e0264138 (2022). 35294956 10.1371/journal.pone.0264138 PMC8926248

[44] Frampton, G. M. et al. Development and validation of a clinical cancer genomic profiling test based on massively parallel DNA sequencing. *Nat. Biotechnol.* **31**, 1023–1031 (2013). 24142049 10.1038/nbt.2696 PMC5710001

[45] Li, H. et al. The sequence alignment/map format and SAMtools. *bioinformatics* **25**, 2078–2079 (2009). 19505943 10.1093/bioinformatics/btp352 PMC2723002

[46] Van Loo, P. et al. Allele-specific copy number analysis of tumors. *Proc. Natl. Acad. Sci. USA* **107**, 16910–16915 (2010). 20837533 10.1073/pnas.1009843107 PMC2947907

[47] Sun, J. X. et al. A computational approach to distinguish somatic vs. germline origin of genomic alterations from deep sequencing of cancer specimens without a matched normal. *PLoS Comput. Biol.* **14**, e1005965 (2018). 29415044 10.1371/journal.pcbi.1005965 PMC5832436

[48] Genomes Project Consortium. A global reference for human genetic variation. *Nature* **526**, 68 (2015). 26432245 10.1038/nature15393 PMC4750478

[49] Alexander, D. H., Novembre, J. & Lange, K. Fast model-based estimation of ancestry in unrelated individuals. *Genome Res.* **19**, 1655–1664 (2009). 19648217 10.1101/gr.094052.109 PMC2752134

[50] Browning, B. L., Zhou, Y. & Browning, S. R. A one-penny imputed genome from next-generation reference panels. *Am. J. Hum. Genet.* **103**, 338–348 (2018). 30100085 10.1016/j.ajhg.2018.07.015 PMC6128308

[51] Browning, S. R. & Browning, B. L. Rapid and accurate haplotype phasing and missing-data inference for whole-genome association studies by use of localized haplotype clustering. *Am. J. Hum. Genet.* **81**, 1084–1097 (2007). 17924348 10.1086/521987 PMC2265661

[52] Stead, L. A. et al. Triple-negative breast cancers are increased in black women regardless of age or body mass index. *Breast Cancer Res.* **11**, 1–10 (2009). 10.1186/bcr2242 PMC2688946 19320967

