# Supplementary material for: Association between ancestry and tumor somatic mutations in a large national cohort of women with breast cancer
Source: NPJ Breast Cancer. 2025 Oct 29;11:117. doi: 10.1038/s41523-025-00815-x (PMC12572180; doi:10.1038/s41523-025-00815-x)
Supplement: Supplementary file 1 — Supplementary Information [file 41523_2025_815_MOESM1_ESM.pdf]

Supplemental Figure 1: Principal component analysis using 1000 genomes reference samples of ancestry in Foundation Medicine Cohort

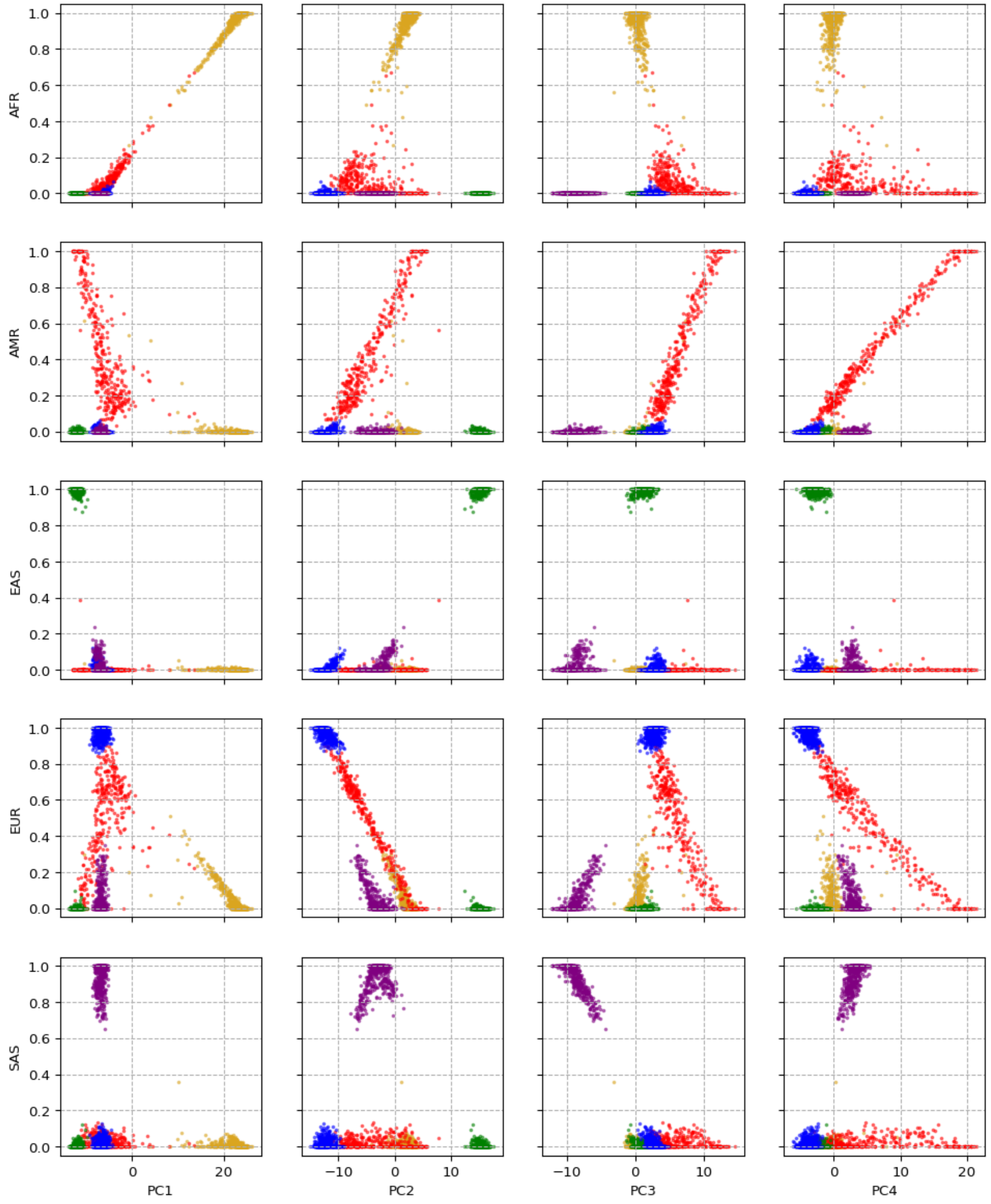

Legend: Principal component analysis using 1000 genomes reference samples of ancestry in Foundation Medicine Cohort. PC=principal component; AFR=African ancestry; AMR=Indigenous American ancestry; EAS=East Asian ancestry; EUR=European ancestry; SAS=South Asian ancestry.

Supplemental Figure 2: Final regression equation for a multi-gene predictor of ER-status

$$p = \frac{e^{\beta_0+\beta_1 X_1+\beta_2 X_2+\beta_3 X_3+\beta_4 X_4+\cdots+\beta_{16} X_{16}}}{1+e^{\beta_0+\beta_1 X_1+\beta_2 X_2+\beta_3 X_3+\beta_4 X_4+\cdots+\beta_{16} X_{16}}}$$

**Term**	**Coefficient**
Constant	β_0 = 2.119696
X_1 = *TP53*_mut	β_1 = -2.06214
X_2 = *GATA3*_mut	β_2 = 1.196157
X_3 = *RB1*_mut	β_3 = -1.221619
X_4 = *PIK3CA*_mut	β_4 = 0.7288263
X_5 = *PIK3R1*_mut	β_5 = -1.697564
X_6 = *CCND1*_cna	β_6 = 0.4844197
X_7 = *RB1*_cna	β_7 = 1.25842
X_8 = *CCNE1*_cna	β_8 = -0.9885205
X_9 = *MAP3K1*_mut	β_9 = 0.6147936
X_{10} = *ATRX*_mut	β_{10} = -0.9860027
X_{11} = *FGFR1*_cna	β_{11} = 0.3853453
X_{12} = *SOX2*_cna	β_{12} = -0.6193694
X_{13} = *NOTCH2*_cna	β_{13} = -0.6535985
X_{14} = *SMARCA4*_mut	β_{14} = -1.712446
X_{15} = *MAP2K4*_mut	β_{15} = 1.712698
X_{16} = *ESR1*_mut	β_{16} = 3.094583

Legend: Multi-gene predictor model for estrogen receptor status with final somatic mutations used in the model and β-coefficients.

Supplementary Data 1a: Short variant mutations associated with African ancestry

Supplementary Data 1b: Short variant mutations associated with Indigenous American ancestry

Supplementary Data 1c: Short variant mutations associated with East Asian ancestry

Supplementary Data 1d: Short variant mutations associated with European ancestry

Supplementary Data 1e: Short variant mutations associated with Southeast Asian ancestry

Supplementary Data 2a: Tumor somatic copy number alterations associated with African ancestry

Supplementary Data 2b: Tumor somatic copy number alterations associated with Indigenous American ancestry

Supplementary Data 2c: Tumor somatic copy number alterations associated with East Asian ancestry

Supplementary Data 2d: Tumor somatic copy number alterations associated with European ancestry

Supplementary Data 2e: Tumor somatic copy number alterations associated with Southeast Asian ancestry

Supplementary Data 3: Association between somatic EGFR mutations and East Asian ancestry
